# Clinical Trials of Fetal Therapy With Continuing Neonatal Interventions: Legal Requirements and Customary Procedures Regarding Parental Consent. *The BOOSTB4 Trial as a Case Study*

**DOI:** 10.1002/pd.70012

**Published:** 2025-10-31

**Authors:** Esther J. Oldekamp, Martine C. de Vries, Anna L. David, Oliver Semler, Magnus Westgren, Cecilia Götherström, E. J. T. (Joanne) Verweij

**Affiliations:** ^1^ Department of Medical Ethics and Health Law Leiden University Medical Centre Leiden the Netherlands; ^2^ Department of Pediatrics Leiden University Medical Centre Leiden the Netherlands; ^3^ Elizabeth Garrett Anderson Institute for Women's Health University College London London UK; ^4^ Department of Pediatrics Faculty of Medicine and University Hospital Cologne University of Cologne Cologne Germany; ^5^ Department of Clinical Science, Intervention and Technology Division of Obstetrics and Gynecology Karolinska Institutet Stockholm Sweden; ^6^ Department of Obstetrics Division of Fetal Therapy Leiden University Medical Centre Leiden the Netherlands

## Abstract

**Objective:**

To analyze the legal consent requirements and customary procedures in clinical trials of fetal therapy with continuing neonatal interventions across four European countries using the BOOSTB4 trial as a case study.

**Methods:**

The study of country‐specific legal consent requirements and the actual consent procedures in the BOOSTB4 trial, incorporating surveys with the trial's principal investigators.

**Results:**

Prenatal consent was obtained solely from the pregnant women in all participating countries, in line with legal requirements. However, in all countries both prospective parents were engaged in prenatal counseling. In Sweden, the Netherlands and Germany obtaining consent from both parents for continuing neonatal interventions is mandatory. In the United Kingdom, officially only the consent of one parent is required. Nevertheless, researchers there are cautious of including pregnant women or neonates if parents disagree to trial participation, a concern echoed by Dutch, German and Swedish researchers.

**Conclusion:**

While the pregnant woman's autonomy is paramount for prenatal trial participation, trials involving continuing neonatal interventions should adopt a two‐step prenatal counseling approach. This approach allows a distinction between consent for fetal and neonatal procedures and allows researchers to comprehend parental perspectives without undermining the pregnant woman's individual rights.

## Key Points

- What is already known about this topic?
  ◦ Pregnant women provide consent for themselves and their fetus.
  ◦ Legal consent requirements for neonatal interventions vary across Europe.
- What does this study add?
  ◦ We reviewed legal consent requirements and customary procedures at planned BOOSTB4 trial sites, focusing on trials with neonatal continuation.
  ◦ For such trials, we propose the following counseling approach: counseling of the pregnant woman first, followed by counseling of both parents to ensure consent for neonatal interventions.

## 1 Introduction

Clinical trials of fetal therapy pose ethical and legal challenges in decision‐making involving the pregnant woman, the fetus, the (future) neonate and potentially the other parent. Legal requirements regarding consent can change throughout the trial, especially when the neonate's continued participation in procedures and/or interventions within a follow‐up study is involved. In line with human rights standards, the EU Clinical Trials Regulation on informed consent [1], and international legal and ethical guidelines, obtaining a pregnant woman's informed consent is a fundamental requirement at the start of clinical trials of fetal therapy across Europe [2]. However, requirements for parental consent for the neonate's ongoing participation can differ between countries [3].

In 2015, the European Network of Pediatric Research gathered data on legal requirements for parental consent across 26 European countries. Their findings indicate that most European countries require both legal parents to consent for their child's participation in a trial [4]. In clinical trials of fetal therapy within these countries, both parents must agree to the neonate's continued follow‐up trial involvement. However, in some countries, such as the United Kingdom and Hungary, only one legal parent's consent is required for a neonate's continued trial participation [4]. From a legal perspective, the pregnant woman can independently decide on her own participation and, after birth, on the neonate's ongoing involvement. This requirement also allows the other parent to approve the neonate's continued participation, even if the mother withdraws consent.

The BOOSTB4 trial (EudraCT 2015‐003699‐60, EUCT: 2023‐504593‐38‐00, ClinicalTrials.gov NCT03706482) is a relevant case study in this context. The BOOSTB4 trial was first approved in 2019. The first participant was included in 2020 and the last participant was included in 2022. The trial involved administering stem cells to fetuses, with continued doses given to the neonate. The objective was to evaluate the safety of multiple intravenous doses of fetal mesenchymal stem cells, as well as the efficacy (including fracture rate and growth) of children with severe Osteogenesis Imperfecta (OI) [5, 6]. A prenatal dose was likely to be more beneficial to the future child's health when followed by postnatal doses [7] underscoring the importance of parental consent throughout the trial. In this paper, we conduct a comparative analysis of the legal requirements and customary procedures related to parental consent in clinical trials of fetal therapy across four countries involved in the BOOSTB4 trial: the Netherlands, the United Kingdom, Sweden and Germany. Each of these countries has its own national legal frameworks, making them relevant for legal‐comparative analysis. We address the following questions:

- Is it legally mandatory to involve both prospective parents in decision‐making during pregnancy regarding the neonate's continued participation in the clinical trial of fetal therapy?
- Must both parents consent for the neonate's ongoing involvement?
- Do researchers engage both (prospective) parents in counseling during and after pregnancy?
- In cases of parental disagreement, do researchers include pregnant women and/or neonates in the trial?

Our focus is on (prospective) parents who have, or will have, the legal authority to act for the child. By “legal authority” we mean individuals who hold or are expected to hold custody or legal guardianship. Throughout this paper, we refer to them as “(prospective) parents”. We analyzed actual practices by conducting open‐ended surveys sent to the principal investigators (PI) of the BOOSTB4 trial (Appendix A) and by reviewing participant information folders and consent forms of the BOOSTB4 trial in the participating countries. The following sites participated: Leiden University Medical Center in the Netherlands, University College London Hospitals in the United Kingdom, Karolinska University Hospital in Sweden, and University of Cologne Children's Hospital in Germany. For the legal and ethical reflection, we examine how involving both parents in counseling and potential parental disagreement affects a pregnant woman's autonomy and individual interests.

## 2 Results: Legal Requirements and Common Practices

With the Clinical Trials Regulation (EU CTR 536/2014), the European Union set out a legally binding framework for clinical trials in the Netherlands, Sweden and Germany, including specific provisions regarding informed consent [8]. While national legislation must comply with this regulation, countries may implement stricter requirements. The transition period of the CTR partially coincided with the BOOSTB4 trial, providing the legal landscape during the trial [8]. Post‐Brexit, the United Kingdom no longer follows European regulations. Below, we explore the current specific legal requirements and common practices related to consent for clinical trials of fetal therapy by answering questions A‐D for the Netherlands (2.1), the United Kingdom (2.2), Sweden (2.3), and Germany (2.4).

### 2.1 The Netherlands

- In the Netherlands, consent for fetal interventions in clinical trials of fetal therapy is regulated by the Dutch Embryo Act. This act demands written consent from the pregnant woman [9] and does not recognize the other prospective legal parent as a participant in decision‐making during pregnancy.
- The Dutch Medical Research With Humans Act mandates that both legal parents' consent for the subsequent neonatal interventions (including postnatal data analysis) [10].
- According to the Dutch PI, involvement of both prospective parents in decision‐making during pregnancy is common. This is reflected in the Dutch prenatal consent forms for the BOOSTB4 trial, which include an optional signature area for the non‐pregnant parent's signature. Researchers often seek proxy consent from both prospective parents for the anticipated neonatal involvement and obtain re‐consent after birth. The postnatal forms used in the BOOSTB4 trial reflect this, with two signature areas labeled as “parent” or “guardian”’.
- While the pregnant woman's consent is sufficient for prenatal participation, the PI may exclude her from trial participation if the prospective parents disagree about continued neonatal involvement. Exclusion may occur if the PI determines that interventions would only benefit the future child if followed by neonatal interventions, or if the trial's outcomes depend on data collected postnatally. Should such disagreement arise in the BOOSTB4 trial, the PI assesses whether prenatal MSC‐injections alone would sufficiently benefit the future child to justify prenatal participation without neonatal involvement. Postnatal parental disagreement excludes the neonate from trial participation.

### 2.2 The United Kingdom

- In the United Kingdom, clinical trials involving Investigational Medicinal Products (CTIMPS) are governed by the Medicines for Human Use Clinical Trials Regulation (2004) (CTR)—not to be confused with the similarly named EU legislation. The BOOSTB4 trial falls under the scope of this regulation. The CTR requires written consent from the pregnant woman who participates in this trial, and it does not address the involvement of the non‐pregnant parent in the decision‐making process [11].
- For continued participation of the neonate in a trial involving CTIMPS, such as the BOOSTB4 trial, the CTR requires consent from a legal parent (or other legally authorized person) [12]. The CTR does not require the consent of both legal parents, which also applies to clinical trials of fetal therapy not involving CTIMPS, as common law does not specifically require double parental consent [13].
- According to the British PI, researchers engage with both (prospective) parents during the prenatal and postnatal decision‐making process, addressing the pregnant woman's participation and the continued involvement of the neonate in trial procedures after birth. The prenatal and postnatal consent forms used for the BOOSTB4 trial in the United Kingdom reflect this shared parental involvement. The prenatal form includes signature areas for the pregnant woman and an optional signature space for the non‐pregnant parent, while the postnatal re‐consent form provides space for the other parents' signature.
- In cases of parental disagreement, the PI states that researchers have concerns about including the pregnant woman in the trial or continuing the trial with the neonate. According to the PI, both parents have parental responsibility once the child is born. Participation by a pregnant woman while the other parent disagrees could potentially place the pregnant woman in a difficult position for the rest of her pregnancy and after the birth, and it could imply conflict between the parents.

### 2.3 Sweden

- In Sweden, the Medicinal Product Act (2015) (Läkemedelslagen) and the Ethical Review Act (2003) (Lag om etikprövning av forskning som avser människor) govern consent in medicinal product trials involving minors or adults. These acts demand written consent from pregnant women when participating in a clinical trial of fetal therapy. The involvement of the non‐pregnant parent in decision‐making is not addressed.
- Both parents should provide consent for continued participation of their neonate in the follow‐up trial. The Ethical Review Act and the Medicinal Product Act require consent by a legal guardian on behalf of the minor [14]. Interpretating these two acts in alignment with the Swedish Parental Code results in the requirement of double parental consent for continued participation in the follow‐up trial [14].
- According to the Swedish PI, both prospective parents are involved in decision‐making during pregnancy. This is reflected in the Swedish BOOSTB4 trial's prenatal consent form, which includes signature spaces for both the pregnant woman and the non‐pregnant parent. After birth, the PI engages with both parents to obtain their re‐consent for continuing neonatal interventions. The postnatal consent form for the BOOSTB4 trial aligns with this practice by providing signature spaces for both parents.
- In cases of parental disagreement regarding the participation of the pregnant woman and continued participation of the neonate, Swedish researchers typically prioritize the wishes of the pregnant woman. However, for the BOOSTB4 trial, pregnant women would not have been included if there was parental disagreement, as continued neonate involvement in trial interventions and procedures requires double parental consent. If parental disagreement arises after birth, the neonate will not continue trial participation.

### 2.4 Germany

- In Germany, the Medical Products Act 2005 (Arzneimittelgesetz, AMG) regulates consent for clinical trials of fetal therapy involving medicinal products. The BOOSTB4 trial falls under the scope of the AMG, which mandates written consent by the pregnant woman [15]. There are no requirements on involving the other prospective parent in prenatal decision‐making.
- When it comes to consent for continued neonatal trial participation, the AMG together with the German Civil code require the legal representatives to consent, which indicates both legal parents [16].
- According to the German PI, it is common to seek consent from both legal parents for fetal interventions as well as for continued neonatal interventions. This preference also exists for clinical trials of fetal therapy where all trial‐related procedures are completed before birth, although legally only the pregnant woman's consent is required. The BOOSTB4 trial was not approved in Germany and hence, information and consent forms were not reviewed by the German authorities.
- In case of parental disagreement, German researchers will not include pregnant women in clinical trials of fetal therapy that begin prenatally and require continued postnatal participation to benefit the future child's health. In cases of postnatal parental disagreement regarding the neonate's continued trial involvement, researchers will engage in discussions with parents but will exclude the neonate if disagreement persists.

## 3 Discussion

Our findings indicate that the legal requirements across the four countries are generally clear and straightforward. For prenatal involvement, the pregnant woman's consent is required. In contrast, continued postnatal involvement of the neonate in trial procedures/interventions mandates double parental consent in the Netherlands, Germany, and Sweden, but not in the United Kingdom. This implies that for clinical trials of fetal therapy starting prenatally and continuing with interventions and/or procedures postnatally, the legal requirements for consent change after birth and vary by country.

However, we also found that the practical implementation of consent procedures does not follow national legal requirements but is more elaborate and comparable across the four countries. Despite not being legally imposed, it is common practice in all countries to involve the non‐pregnant parent in the counseling process regarding the pregnant woman's participation. In the BOOSTB4‐ consent form, both prospective parents were sought during pregnancy and for the continued participation of the neonate—even in the United Kingdom, where the legal requirement for neonatal participation is single‐parent consent.

Minor differences exist between countries regarding the inclusion of the pregnant woman when researchers are aware of parental disagreement about the neonate's continued involvement. In the United Kingdom, the PI would disclose a general refusal, whereas in other countries, the decision depends on the specific trial and a risk‐benefit analysis, considering whether the future child would benefit sufficiently solely from prenatal involvement.

Current clinical trials of fetal therapy may increasingly involve continued neonatal interventions/procedures, as seen with prenatal and postnatal stem‐cell doses in the BOOSTB4 trial. This type of fetal therapy research is different from previous research where interventions focused for for example on pregnancy related diseases, or involved fetal interventions exclusively, such as laser therapy for complex twin complications or intra‐uterine fetal blood transfusion without continued neonatal interventions [17].

In our view, fetal therapy researchers need to solicit the opinions of both parents regarding the anticipated neonatal involvement to properly balance the risks and benefits associated with prenatal participation. When fetal participation is likely to be more or only beneficial to the future child's health when followed by postnatal participation (as in the BOOSTB4 trial), potential dissent from one parent regarding continuing neonatal interventions impacts the risk‐benefit analysis, making the trial (almost) futile for that individual child, and preventing the full outcome analysis that such a trial will require. This underscores the importance of monitoring parental opinions throughout the trial. Conversely, when there are clear fetal or health benefits for the future child resulting from trial participation by the pregnant woman (e.g., in pregnancy related diseases), the risk‐benefit equilibrium, combined with her autonomy, may justify dismissing the other prospective parent's objections regarding continuing neonatal interventions. In cases where continuing neonatal participation would only involve observational follow‐up and data gathering, disagreement between parents would only disadvantage the trial results.

To address the above‐mentioned concerns for trials where interventions and/or procedures begin in the fetus and continue in the neonate, we propose a prenatal counseling procedure that consists of two separate sessions with two different information and consent forms: one for the pregnant woman and another for both prospective parents. For consistent practice, we recommend integrating this procedure into professional guidelines. However, before such integration, we recommend exploring the counseling procedure in a focus group study, using a hypothetical or future planned fetal therapy trial with continuing neonatal interventions, to gather additional input from practitioners (neonatologists and fetal medicine specialists) and prospective parents. This focus group study would ensure interdisciplinary input and reflection, while also supporting consensus‐building. With this additional input, a more comprehensive understanding of the separate counseling procedure can be reached. Below, we elaborate on our proposed separate counseling sessions.

### 3.1 Two Separate Counseling Sessions: Of the Pregnant Woman and the (Prospective) Parents

The pregnant woman should separately consent for herself as a research participant and as the parent of the (future) child. The maternal‐fetal interventions directly affect her bodily integrity, and after birth she will be the neonates legal guardian. With two separate counseling sessions, there will be focused attention on protecting the pregnant woman’s autonomy in one session, while the second session will focus on her future role as the neonate's legal parent, along with the corresponding parental rights and responsibilities.

During the session focused on the pregnant woman's participation, she will be the primary conversation partner and her interests will be the prime consideration. Focusing on the pregnant woman's involvement in this part of the counseling procedure may help ensure that her voluntariness is respected, identify possible signs of inequality between prospective parents, and provide an opportunity to specifically address her individual health interests in a dedicated setting. This approach aligns with the principle that an adult's informed consent for research participation should aim to safeguard bodily integrity, ensure voluntariness, and protect privacy [18]. We recommend researchers to speak with the pregnant woman alone, at least for a while.

When the pregnant woman consents to participate in the study, a separate second counseling session is initiated. In this session, researchers counsel both prospective parents, addressing the continuing neonatal interventions and/or procedures and how postnatal participation relates to prenatal involvement. This step anticipates the transition from fetus to neonate, who becomes a legal subject with independent rights after birth. As the neonates cannot yet decide for themselves, the parents are required to provide (proxy) consent. This counseling step provides researchers with insight into parental opinions and prepares parents for their future legal responsibility. Proxy consent can be obtained when parents agree. However, dual parental re‐consent after birth remains a legal obligation in Sweden, the Netherlands and Germany.

During this second counseling step, researchers can discuss potential parental disagreements together with the parents. When disagreement persists, researchers could present and discuss the option for the pregnant woman to participate in the prenatal part of a trial that involves fetal interventions with a clear prospect of direct benefit to the fetus or future child, without the need for neonatal interventions/procedures. However, this will need to be acceptable to the clinical trial team and be a recognized option within the approved trial protocol before the trial starts. This approach may help avoid conflicts between parents in cases where the pregnant woman proceeds without the other parent's agreement. If parental disagreement persists, one parent may seek judicial authorization to substitute for the consent of the non‐consenting parent, provided such substitution is permitted under the trial protocol.

Separate counseling ensures that pregnant women feel safe during counseling, but by including both prospective parents our counseling approach also builds on the ethical considerations raised by Johansson et al. in their paper on the possible requirement for paternal (or partner) consent alongside maternal consent in prenatal research [19]. Johansson et al. examined the father's role in clinical trials of fetal therapy and considered the possibility of paternal consent alongside maternal consent. While they acknowledge the value of paternal input and the overlap of interest between parents, they also acknowledge the tension this could create with the pregnant woman's rights.

### 3.2 Two Separate Informed Consent Forms: One for the Pregnant Women and One for the (Prospective) Parents

When counseling results in agreement regarding the pregnant woman participation and/or continued involvement of the neonate, informed consent forms should be signed. We recommend using separate participant information and informed consent forms. The form for the pregnant woman's participation should specifically and exclusively address her as the participant who will be signing the form. The non‐pregnant parent should not be included on the form as a co‐signatory. In contrast, the post‐birth re‐consent form regarding the continued neonatal involvement in trial interventions/procedures should include both parents in countries where dual parental consent is required.

When two parents are present, our proposed two‐step prenatal counseling procedure offers valuable insights and allows for dual proxy consent. However, it is important to acknowledge that parental disagreement could arise later, for example, during the re‐consent process. Nevertheless, our suggestion can still ensure researchers to be timely informed of potential parental disagreement during the fetal phase of a trial in a manner that safeguards the pregnant woman's individual interests and allows for discussion with disagreeing parents.

We have to acknowledge that the findings of the surveys are views of the researchers answering the survey, and do not represent the views of “all” researchers in the fetal therapy field. Moreover, the study was limited to four European countries, while the concerns we have identified could also be relevant in countries such as the United States. It was not possible to examine actual clinical practice because the BOOSTB4 trial did not proceed.

## 4 Conclusion

Pregnant women's autonomy in clinical trials of fetal therapy is paramount. For trials in which fetal interventions/procedures will continue in the neonate, we propose a two‐step prenatal counseling process with separate information and consent forms. Researchers first counsel the pregnant woman alone and then counsel both prospective parents together. After birth, the legally required re‐consent is obtained.

This prenatal counseling approach allows researchers to gain insights into parental opinions, helping them balance risks and benefits when deciding whether to include the pregnant woman in trials with continuing neonatal interventions while safeguarding her autonomy. This approach also provides a safe platform to discuss the woman's individual health interests, especially when she feels unsafe with the non‐pregnant parent. To ensure uniform practices, we recommend integrating this procedure into professional guidelines.

## Funding

This project has received funding from the European Union's Horizon 2020 research and innovation programme under grant agreement No 681045, Vetenskapsrådet (The Swedish Research Council 921‐2014‐7209 and 2020‐01666), Region Stockholm in Sweden (20200365, FoUI‐975495), the Center for Innovative Medicine (CIMED) in Sweden (FoUI‐962010), and the Foundation for Children of the Freemasons in Stockholm. The funding sources played no role in the study design, data collection, data analysis, interpretation of results, or writing of the report.

## Ethics Statement

The authors have nothing to report.

## Consent

The authors have nothing to report.

## Conflicts of Interest

Magnus Westgren and Cecilia Götherström are co‐founders and co‐owners of BOOST Pharma ApS founded in 2020. BOOST Pharma ApS had no involvement in the design, conduct, or reporting of this research. No conflicts of interest declared by the other authors.

## Data Availability

The data that support the findings of this study are available from the corresponding author upon reasonable request.

## Appendix A Survey Actual Practice Parental Consent in Clinical Trials of Fetal Therapy With Continuing Neonatal Interventions

This survey aims to investigate the requirements concerning parental consent in your country. The purpose is not to seek the “legally correct answers”, but rather to explore how legal requirements influence your common practice. How do you implement these legal requirements? The goal is not to examine personal preferences regarding legal requirements.

I. For research of the new‐born:

Is consent required from a single parent or both parents before including the new‐born in a research trial?

How do legal requirements regarding consent by a single parent or by both parents affect your common practice? What happens if one of the parents does not agree with participating in the trial?

II. For maternal‐fetal interventions:

Is consent required from the pregnant women only or both future parents before including the pregnant woman in a fetal therapy research trial?

How do legal requirements regarding consent affect your common practice? What happens if the other future parent does not agree with participating in the trial?

III. For trials that extend from prenatal to postnatal:

Is additional consent required from the other future parent before including pregnant women in fetal therapy trials that start prenatally and continue postnatally?

How does this requirement affect your common practice?

When do you sign the ICF?

Would you include a pregnant woman in a fetal therapy trial if she consents, but the other future parent disagrees with the post‐birth interventions? What factors would influence your decision?
